# “She’s my memory; and he’s my legs!” The experiences of couples living with dementia and multiple health conditions

**DOI:** 10.1002/alz.086416

**Published:** 2025-01-09

**Authors:** Rosie J Dunn, Emma Wolverson, Andrea Hilton

**Affiliations:** ^1^ University of Hull, Hull, East Yorkshire United Kingdom; ^2^ Dementia UK, London, London United Kingdom; ^3^ University of Hull, Hull United Kingdom

## Abstract

**Background:**

Dementia rarely travels alone. People with dementia (PwD) often have multiple other physical diagnoses (multimorbidity) and experience poor quality, fragmented care. Over two thirds of carers of PwD are spouses, over half of which are 85 years old or above. Age is strongly associated with increased multimorbidity; therefore, carers are also likely to experience multimorbidity. Consequently, married or cohabiting couples are likely to experience the dementia journey together as well as live with multimorbidity. However, research has neglected to explore couples’ experiences of living with both dementia and multimorbidity, and the potential challenges they may face. Understanding couples’ experiences collectively will inform the development of appropriate, psychosocial interventions.

**Method:**

Married couples aged 65 or above were asked to keep a photo diary for one month, taking photographs of their experiences of living with dementia and multimorbidity. Couples were given a choice to use either disposable cameras or their own digital device. This was followed by an audio‐recorded interview, using their printed photographs as a photo‐elicitation technique to aid discussion. Interpretative Phenomenological Analysis (IPA) was used to analyse the data.

**Results:**

Six couples participated. IPA revealed couples have to ‘fight for help’, particularly in relation to dementia and stroke care; living with uncertainty was a significant theme. Couples discussed changes in their relationship, and behavioural and practical adaptations they have made in order to support each other. Some couples do not identify as being ‘ill’ or ‘disabled’ despite the multimorbidity they experience. Maintaining hobbies and interests that are important to them both as a couple and individually was vital for them to continue living a good quality of life.

**Conclusion:**

Couples living with dementia and multimorbidity face significant challenges that can pose a threat to their relationship and quality of life. Challenges include inadequate support and healthcare, changes in the relationship and couple identity. However, despite this, couples maintain a positive outlook and ‘work as a team’ in order to adapt to each other’s health conditions and to continue leading a fulfilling life. These findings can be used to develop relationship‐centred interventions to support couples in the community.

